# Early Warnings: The Lessons of COVID-19 for Public Health Climate Preparedness

**DOI:** 10.1177/0020731420928971

**Published:** 2020-07

**Authors:** Mary C. Sheehan, Mary A. Fox

**Affiliations:** 1 Department of Health Policy and Management, Johns Hopkins Bloomberg School of Public Health, Baltimore, Maryland, USA

**Keywords:** COVID-19, climate change, public health

## Abstract

The early 2020 response to COVID-19 revealed major gaps in public health systems around the world as many were overwhelmed by a quickly-spreading new coronavirus. While the critical task at hand is turning the tide on COVID-19, this pandemic serves as a clarion call to governments and citizens alike to ensure public health systems are better prepared to meet the emergencies of the future, many of which will be climate-related. Learning from the successes as well as the failures of the pandemic response provides some guidance. We apply several recommendations of a recent World Health Organization Policy Brief on COVID-19 response to 5 key areas of public health systems – governance, information, services, determinants, and capacity – to suggest early lessons from the coronavirus pandemic for climate change preparedness. COVID-19 has demonstrated how essential public health is to well-functioning human societies and how high the economic cost of an unprepared health system can be. This pandemic provides valuable early warnings, with lessons for building public health resilience.

If there were doubts about the need to urgently strengthen public health systems worldwide to prepare for climate change, the coronavirus disease 2019 (COVID-19) pandemic has dispelled them. The early 2020 response to COVID-19 revealed major adaptive capacity gaps as public health systems around the world were overwhelmed by this quickly-spreading new virus.^1–4^ While there is currently no evidence the SARS-CoV-2 coronavirus that causes COVID-19 is associated with climate change,^5^ researchers have been concerned for decades about the potential for global warming to support conditions for infectious disease outbreaks and to amplify other health impacts associated with heat, drought, storms, and related hazards.^6,7^ Prominent voices have urged building health system preparedness and resilience to these climate-related threats.^6,8,9^ Yet chronic underfunding has plagued national and local public health agencies across many parts of the world.^4,10^ Only 5% of international spending for climate adaptation is directed toward public health,^11^ and health agencies are often poorly integrated into climate change planning.^12,13^ While the critical task at hand is turning the tide on COVID-19, this pandemic serves as a clarion call to governments and citizens alike to ensure public health systems are better prepared to meet the emergencies of the future, many of which will be climate-related.

Climate change threatens population health in multiple ways. In 2018, 220 million more older adults were exposed to heatwaves, a top cause of direct climate-related deaths, than in 2000.^11^ Climate change also affects health indirectly, through interaction of climate hazards with social, natural, or built environments.^14^ One example is dengue fever: with changes in heat and humidity, its vectorial capacity has increased worldwide by up to 10% since the 1950s.^11^ A further concern is the potential for multiple impacts to co-occur, stretching coping capacity; a timely illustration is concern about flood or hurricane seasons coinciding with peak coronavirus cases in certain locations.^15^ Risks related to these direct and indirect climate impacts are increasing around the world; they are becoming particularly acute in Asia and Africa and in urban areas more generally.^16^ The 2018 “Adaptation Gap Report” of the United Nations Environment Program focused on health and identified several of the main challenges: building health system skills, workforce, and funding; placing greater attention on economic and social factors that lead to climate-relevant disease; and enhancing use of climate-adaptive tools such as early warning systems, and enhanced surveillance.^17^ Using the framework for building blocks of resilient health care systems defined by the World Health Organization (WHO),^8^ these needs can be summarized in 5 themes: governance arrangements; information and communications; core health services; determinants of health and disease; and capacity, research, and training of workers in health fields.

How can the lessons of COVID-19 be applied to filling these public health gaps to meet the challenges of climate-related risks to population well-being? Learning from the successes as well as the failures of the pandemic response provides some guidance. Based on knowledge and experience with the pandemic thus far, the WHO has issued a Policy Brief recommending ways to strengthen European health systems to respond to COVID-19.^3^ Below, we apply several of these recommendations to the 5 climate-related public health system gap areas to suggest some early lessons from the coronavirus pandemic.

## Governance

Clear roles and responsibilities are essential to well-functioning public health systems. The response to COVID-19 in many countries revealed institutional confusion across agencies. In the United States, for example, the rollout of diagnostic testing and the efforts to ensure hospitals had personal protective equipment and ventilators demonstrated miscommunication and unclear mandates among large federal agencies such as the Centers for Disease Control and Prevention (CDC), Food and Drug Administration, Federal Emergency Management Agency, and state public health departments, hospitals, private laboratories, and equipment suppliers; this confusion resulted in lost time and lives.^18–20^ The WHO Policy Brief recommends actions that aim to avoid such pitfalls, including reviews of supply chains, assembling stocks of essential medicines and health technologies, and ensuring clarity in roles, relationships, and coordination mechanisms in health system governance.^3^

Climate-driven health risks have already begun to challenge public health and health care systems in similar ways, including the challenges of sourcing, stockpiling, and distributing emergency equipment and of scaling up hospital surge capacity. An example is the shortages of N-95 masks during the intense “smoke-waves” caused by the 2018–2019 California wildfires and 2019–2020 Australia bushfires.^21,22^ Climate change is creating the need for clearer coordination mechanisms in other ways also. This includes defining public health relationships with partner agencies – for example, with urban planning departments for “greening” initiatives to reduce the urban heat island effect, or with water utilities for demand management in times of drought – as identified in Barcelona.^23^ Another example is the need to coordinate with electric power utilities to ensure hospital storm preparedness and post-disaster response, as found in New York after Superstorm Sandy.^24^ Whether between government levels, across government agencies, or with private service providers and stakeholders, a lesson from the COVID-19 pandemic is that addressing health challenges from climate change will require redoubling efforts to define clear modes of collaboration among actors with responsibilities that affect health outcomes in a crisis.^25^

## Information

Risk communication to the public is essential in a health emergency. South Korea’s early management of the COVID-19 pandemic has been looked to as a model of public outreach. Legislation passed after the 2003 SARS outbreak defined roles for government and private actors that emphasized transparency and frequent communication; under this law, the government and the Korean Centers for Disease Control and Prevention quickly responded to COVID-19 with strong public messaging on hand washing and social distancing, twice-a-day press briefings, regularly updated online information, and targeted text messages.^26^ Examples of intensive health information-sharing have also evolved in many jurisdictions globally. Health departments around the world have communicated detailed social-distancing guidance and provided daily updates on COVID-19 cases, hospitalizations, and deaths, often broken down by age, gender, and other variables. While much is still unknown about this coronavirus, individuals have an unprecedented level of health-related information available on what is known regarding COVID-19 risk reduction and local evolution of the pandemic. National and global mapping initiatives have also sprung up, including the Johns Hopkins Center for Systems Science and Engineering COVID-19 map, now a relied-upon source of data for the media, researchers, and the public.^27^ Building on such lessons, the WHO Policy Brief recommends expanding communication capacity and proactively managing media relations.^3^

Information-related activities are among the most frequently reported climate adaptation actions by local governments. For example, geographic information system mapping of extreme heat and flood hazards, including vulnerability factors such as absence of air conditioning or lack of greenspace, is already an important tool in the climate resilience arsenal of world cities such as San Francisco and London.^16,28^ This information could be scaled up to address additional risks and locations, and it could be made even more health-relevant by incorporating surveillance and risk data and climate-relevant health monitoring indicators such as geographic distribution of health risks or results of syndromic surveillance.^29^ More regular communication with the public via regularly updated online portals and direct text messaging, including well-targeted information on prevention, diagnostic, and treatment strategies for those at risk, would contribute to better public emergency preparedness and resilience. COVID-19 has demonstrated the importance of health information and shown there is a strong demand for it. Both the public and public health agencies could benefit from amplifying information flow on climate-relevant disease surveillance, risk and hazard information, and strategies, particularly for the most vulnerable.

## Services

Public health agencies are responsible for carrying out a range of services to ensure population well-being, including testing, case reporting, surveillance, and contact tracing – all essential tasks in managing an infectious disease epidemic. Singapore’s early successful response to COVID-19 has been attributed in part to the city’s buildup of these services after the 2003 SARS outbreak, testing of them during the 2009 H1N1 pandemic and 2015 Zika outbreak, and reliance on this experience to guide detection, contact follow-up, and triage using public health clinics to identify those with relevant symptoms.^30,31^ Singapore employed digital contact tracing, which – although accompanied by risks to privacy – has been seen as successful in East Asia generally.^32^ Other jurisdictions are following another approach, one that partly offsets reduced public health services related to budget cuts: for example, Massachusetts has hired a corps of 1,000 contact tracers as an auxiliary to the public health department, whereas Ireland has deployed a similar number of furloughed government workers to the contact tracing task.^33^ The WHO Policy Brief recommends training, mobilizing, and repurposing the health workforce according to priority services.^3^

Climate change similarly challenges the effective delivery of badly-needed public health services. Also similarly, there is potential for public health agencies to engage with digital technology and to train, retrain, and repurpose the workforce – and/or attract a corps of volunteers – to strengthen public health services, while also building community resilience and preparedness. Such auxiliary corps could be allocated to priority public health tasks, depending on locally defined climate hazards. Citizen-science initiatives are already achieving some of these goals: for example, collecting temperature data in areas of cities that may be vulnerable and poorly reflected in regular measurement systems, or documenting locally occurring flooding in poorly mapped, at-risk neighborhoods.^34^ The experience of COVID-19 suggests the opportunity for, and perhaps some public willingness to support, nontraditional approaches to augment core public health service provision in order to address growing risks from climate change impacts on health.

## Determinants

A large literature calls for greater focus on the upstream determinants of health, including the close connection of physical and mental health with environmental, economic, and social well-being.^35^ Restrictive social-distancing measures designed to “flatten the curve” of coronavirus resulted in shutdowns that, in turn, have brought unprecedented economic impacts: Estimates suggest global growth may slow by as much as 2% per month, whereas global trade could fall by 13% to 32%, depending on the pandemic peak and trajectory.^36^ In the United States, unemployment claims had reached 16 million by mid-April 2020.^37^ International Monetary Fund tracking of economic policy responses indicates many countries have taken fiscal measures aimed at easing the strain on households by providing health or unemployment coverage or supporting small businesses affected by shutdowns.^38^ On the other hand, the impacts of COVID-19 have also brought decreased greenhouse gas emissions, with a projected 2020 reduction of 6% in global energy demand, as well as major reductions in transportation.^39^ Cities such as Milan are planning ways to continue the unanticipated health advantages of reduced vehicle traffic, such as adding bike lanes and relying more on active transport beyond the shutdown period.^40^ The WHO checklist highlights the need to optimize social protection to mitigate the impact of COVID-19 policy measures on households.^3^

While not brought about by restrictive public health policies, climate change-related population impacts in many countries and regions are likely to be extensive. Disasters such as hurricanes, floods, and wildfires create risk of injury and loss of life, but also damage to property and assets that may dramatically affect personal financial well-being and mental health. Much of the disaster-related property damage worldwide is uninsured, leaving uncompensated losses and accompanying stress.^11^ One in 6 survivors of Hurricane Katrina were found in interviews to have post-traumatic stress even 12 years after the 2005 storm.^41^ Similarly, among the likely impacts of climate-related drought in vulnerable areas are undernutrition and, in some cases, migration and conflict.^42^ Effective public health policy that takes account of determinants of health will increasingly need to address the social and economic well-being of the most vulnerable, provide for social safety nets, and account for mental health needs. At the same time, the health gains related to links to broader determinants should be maximized: for example, estimating co-benefits to health from actions that result in continued lower greenhouse gas emissions. The ongoing effort to define policies that protect populations from the enormous economic impact of COVID-19 – combined with the reality of reduced emissions – may provide opportunities to identify sustainable social protection strategies for climate-related population health risks.

## Capacity

Public health capacity is grounded in technical skills, data, and knowledge, but also requires an ability to lead implementation of a coherent strategy to protect populations. Hong Kong’s early COVID-19 response provided the world with a lesson in public health leadership. With its 7.5 million population, as of mid-April 2020, Hong Kong had only 4 deaths due to COVID-19. Its strategy included a suite of measures, among them rapid and intensive surveillance, case and contact isolation, strict social distancing, closing of schools, and effective communication.^43^ Several universities and public health agency partners, along with citizen volunteers, are now developing an open database of COVID-19 policies across countries that will include rating the success of various interventions; one example is a COVID-19 policy “stringency index,” at which Hong Kong has excelled.^44^ In this context, the WHO checklist recommends bolstering the capacity of public health organizations and professionals to respond to an emergency.^3^

Similarly strong public health capacity – knowledge, skills, and leadership – will increasingly be needed to manage the health challenges of climate change.^12^ Building more effective capacity to reduce the risks of COVID-19 is likely to also help public health agencies improve their adaptive capacity to address climate-related population health risks, whether heat-related illness, increased childhood asthma, or a host of other climate health risks.^45^ Efforts to build capacity, such as the CDC Climate-Ready States and Cities program^13^ or the Center for Climate Change and Health capacity-building project, have shown what can be done at low cost to train the public health workforce in local jurisdictions.^46^ City networks that share experience, lessons, and training are also a promising strategy.^47^ Partnerships between academia and practitioners to develop shared and disseminated intervention evaluations networked across jurisdictions, as is being done with COVID-19, would be helpful to bolster climate-adaptive public health capacity.^48^ Public health actors have an opportunity to transition from the strong leadership role they have with COVID-19 to an enhanced leadership role within climate change planning and action.

## Conclusion

The experience with COVID-19 provides substantial lessons on health-related adaptive responses to climate change hazards. The challenges of the pandemic and of climate change both require proactive action from public health agencies to protect populations. Climate change will require clearly defined arrangements for responsibilities across actors engaged in protecting the public’s health – whether government jurisdictions, sectoral agencies, or stakeholders. Public health and other agencies may be able to learn from – and adapt to climate change-related health risks – some of the highly responsive, targeted, and frequently updated health information practices emerging with COVID-19. Health agencies will also need to double down on their core functions and may benefit from enlisting the support and engagement of citizen volunteers; among the benefits of such a strategy may be enhanced community preparedness. Interventions in support of climate-relevant health impacts will increasingly have to take into account broader determinants and the full concept of well-being: physical, mental, social, economic, and financial. Finally, the public health field will also need to model adaptive capacity leadership and to work to build the skills required for this, including better integration in broader climate change planning and action. COVID-19 has demonstrated how essential public health is to well-functioning human societies and how high the economic cost of an unprepared health system can be. It provides us with valuable early warnings of future risk in a warmer and less predictable climate. We can build on the best of these adaptive public health responses by mainstreaming them into our climate change strategies to ensure a more resilient future.
